# Management of a primary malignant melanoma of uterine cervix stage IVA patient with radical surgery and adjuvant oncolytic virus Rigvir^®^ therapy: A case report

**DOI:** 10.1002/ccr3.2928

**Published:** 2020-05-22

**Authors:** Elizabete Pumpure, Eva Dručka, Dana Kigitoviča, Raimundas Meškauskas, Sergejs Isajevs, Ineta Nemiro, Agnija Rasa, Evija Olmane, Tatjana Zablocka, Pēteris Alberts, Simona Doniņa

**Affiliations:** ^1^ Department of Obstetrics and Gynaecology Riga Stradiņš University Riga Latvia; ^2^ Riga Maternity Hospital Riga Latvia; ^3^ Department of Internal Diseases Riga Stradiņš University Riga Latvia; ^4^ National Center of Pathology Affiliate of Vilnius University Hospital Santaros Klinikos Vilnius Lithuania; ^5^ Centre of Pathology Riga Eastern Clinical University Hospital Riga Latvia; ^6^ Department of Pathology Faculty of Medicine University of Latvia Riga Latvia; ^7^ Department of Diagnostic Radiology Oncology Centre of Latvia Riga Latvia; ^8^ R&D, Rigvir Riga Latvia; ^9^ Department of Radiology Pauls Stradiņš Clinical University Hospital Riga Latvia; ^10^ Oncology Centre of Latvia Riga East University Hospital Riga Latvia; ^11^ Institute of Microbiology and Virology Riga Stradiņš University Riga Latvia

**Keywords:** ECHO‐7 virus, melanoma, oncology, oncolytic virus, Rigvir, uterine cervix

## Abstract

Primary malignant melanoma of the uterine cervix is a rare disease with poor prognosis and high recurrence rate. We used Rigvir^®^ as adjuvant therapy for a stage IVA patient. Tolerability, overall and progression‐free survival are good.

## Graphical abstract

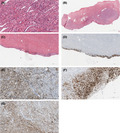

## 1 INTRODUCTION

A 25‐year‐old nulliparous woman was diagnosed with a primary malignant melanoma of the uterine cervix stage IVA. She has undergone surgery three times. The oncolytic virus Rigvir^®^ was prescribed between the second surgery and third surgery. Pembrolizumab therapy is planned. Considering the aggressive diagnosis, the patient shows good progression‐free survival.

The incidence of malignant melanoma has increased over the last 20 years. Genital tract melanomas account for less than 2% of the cases, mostly of vulvovaginal origin, while other gynecologic sites are affected even more rarely; by 2012, only about 78 primary malignant melanomas of cervix have been described. The diagnosis of primary malignant melanoma of the cervix is mostly made in menopausal woman with a median age at diagnosis of 59.0 years. Then, the emphasis of the therapy is on radical surgery but there is no consensus regarding adjuvant treatment and use of chemotherapy, radiotherapy, and immunotherapy or in combination. The overall prognosis is poor, irrespective of the stage at the time of the diagnosis.^1^

The aim of this report is to describe the use of oncolytic virotherapy with Rigvir^®^ as adjuvant treatment after surgery of advanced primary malignant melanoma of uterine cervix stage IVA. A preliminary report after surgery has been published,^2^ before start of Rigvir^®^ virotherapy.

## 2 CASE PRESENTATION

**FIGURE 1:**
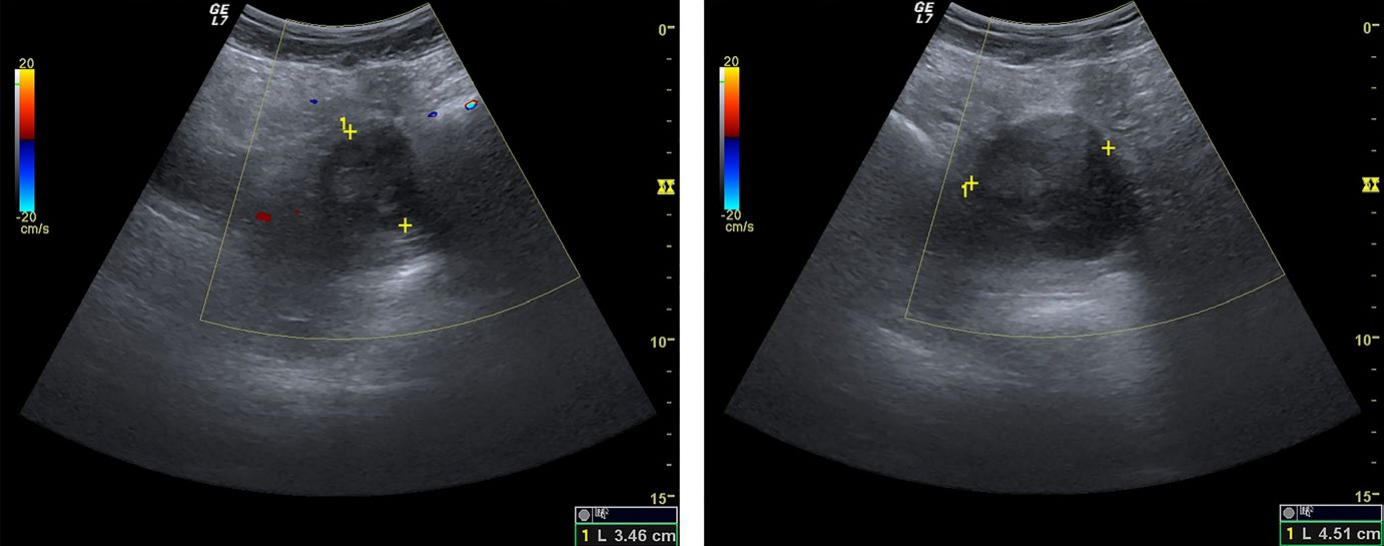
Abdominal ultrasound on 27 September 2014 shows a mass in the pelvis (4.51 × 3.46 cm)

A nulliparous otherwise healthy woman born in 1989 was admitted to the emergency department on 27 September 2014 with her first episode of acute pain in the lower abdomen. Transabdominal ultrasound revealed a mass in the pelvis 4.51 × 3.46 cm (Figure 1), and the patient was discharged and recommended additional examinations in an out‐patient clinic.

Three days later, on 30 September 2014, the patient was repeatedly admitted at the P. Stradiņš University hospital in Riga with the same complaints. During laparoscopy, a soft, oval, 4 cm lesion in the anterior part of the uterus between *plica vesicouterina* and the uterine cervix was found and removed. However, it was not possible to obtain tumor‐free resection margins because of expansion of abnormal tissue to the peritoneum, uterine cavity, and internal cervical orifice.

**FIGURE 2:**
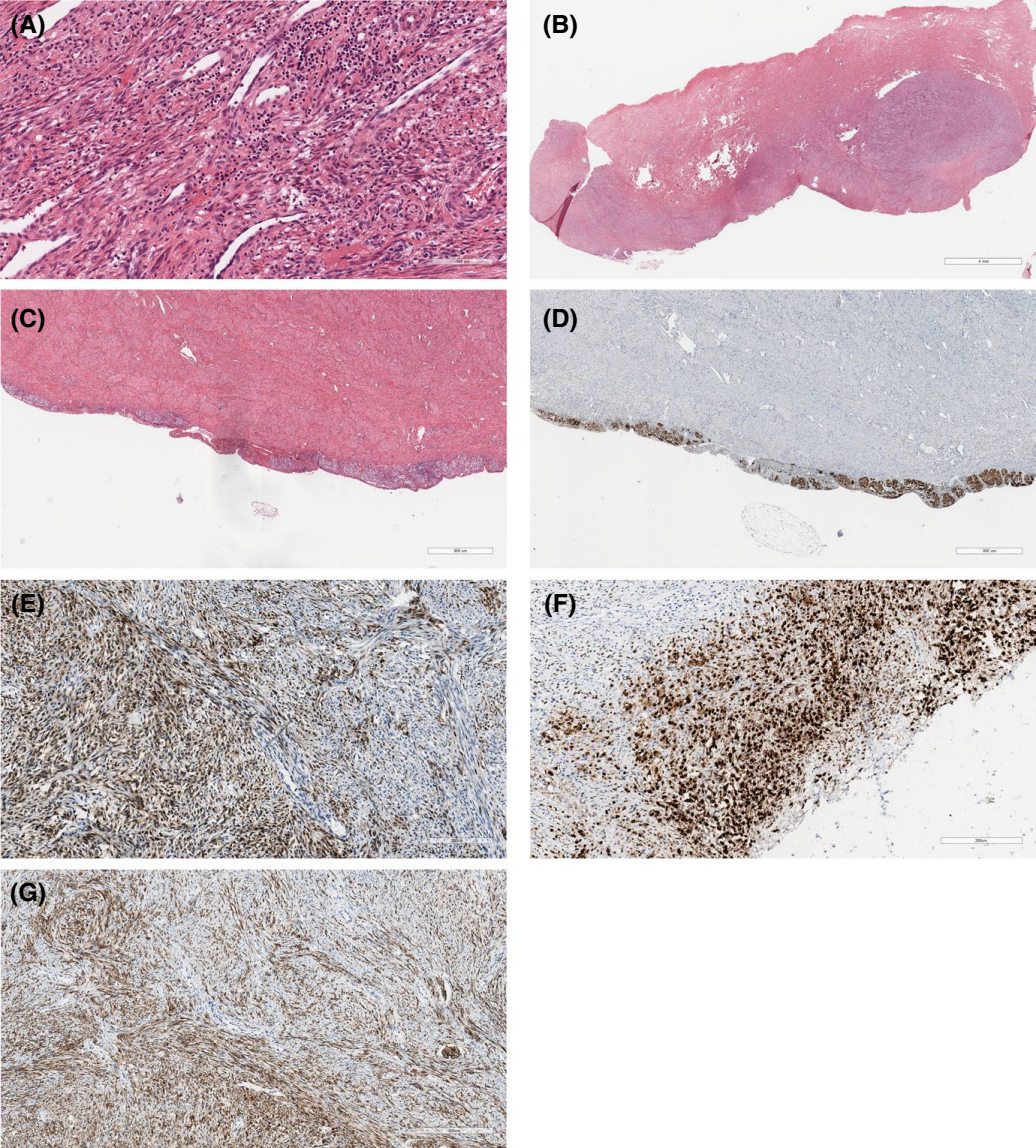
Histological photomicrographs of melanoma of the uterine cervix. (A) Representative photomicrograph showed cervical melanoma, consisted from tumor nests, fascicles, and solid areas of epithelioid and spindle cells. Hematoxylin and eosin stain. Scale bar 100 μm. (B) Representative photomicrograph showed melanoma in the cervical stroma. Hematoxylin and eosin stain. Scale bar 4 mm. (C) Representative photomicrograph showed melanoma in the serosal surface. Hematoxylin and eosin stain. Scale bar 800 μm. (D) Immunohistochemical staining with MelanA. Scale bar 800 μm. (E) Representative photomicrograph showed MelanA immunopositivity in tumor cells. Immunohistochemical staining method. Scale bar 200 μm. (F) Representative photomicrograph showed MITF immunopositivity in tumor cells. Immunohistochemical staining method. Scale bar 200 μm. (G) Representative photomicrograph showed diffuse HMB45 immunopositivity of various intensity in tumor cells. Immunohistochemical staining method. Scale bar 300 μm

The obtained tissue sample was found to contain nests, fascicles, and solid areas of epithelioid and short spindle cells. Tumor cells had clear, focally pigmented cytoplasm with oval and moderately pleomorphic nuclei. The tumor was spread in the stroma and epithelial‐stroma junction of the uterine cervix, measured approximately 25 mm. There were tumor nests in the serosal surface of the uterine corpus and ovary. Immunohistochemistry demonstrated that the tumor cells were positive for MelanA and MITF, diffuse positivity of various intensity for HMB45 and weak reaction for S100 (Figure 2).

**FIGURE 3:**
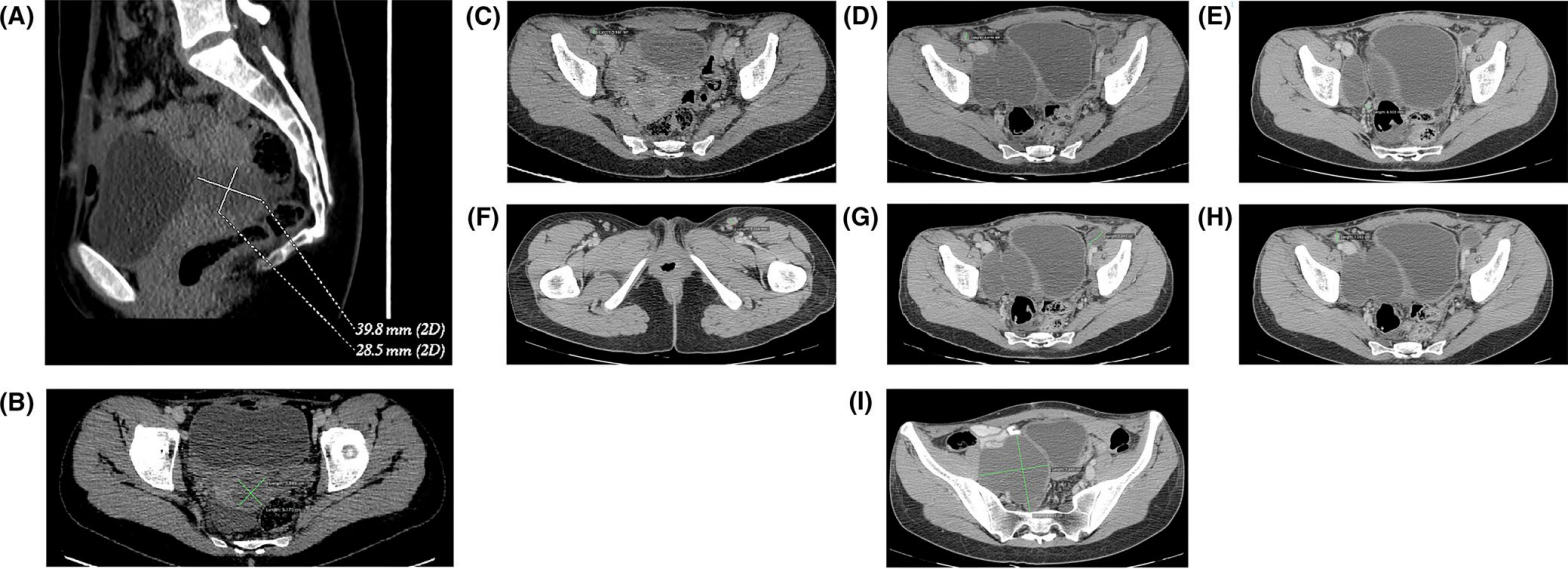
Contrast‐enhanced computed tomography (CT) scans performed on 15 October 2014 and 24 March 2015. (A and B) CT scan performed on 15 October 2014 after the first surgery shows a residual tumor. (C and D) Pelvic CT scan shows lymph node size growth from 0.6 cm (15 October 2014) to 0.9 cm (24 March 2015). (E, F, G, and H) Enlarged lymph nodes with the largest diameter of 2.2 cm (24 March 2015). (I) Bilateral lymphoceles (24 March 2015)

After the surgery, computed tomography (CT) showed a residual tumor in the pelvis measuring between 3.98 × 2.85 cm and 3.56 × 3.17 cm (Figure 3A,B). Assessment for melanotic lesions in the skin, uveal tract, and other mucosal sites was negative. The diagnosis of primary malignant melanoma of the uterine cervix was assigned.

The patient was transferred to the Latvian Oncology Centre of the Riga Eastern Clinical University Hospital for surgery: Radical hysterectomy type II without oophorectomy was performed on 19 November 2014. During the investigation of the abdominal cavity, wide abnormal adhesions between omentum and anterior abdominal wall were noticed, and therefore, partial omentectomy was performed. A total of 17 lymph nodes from the pelvis and 3 enlarged lymph nodes from the omentum were removed and histologically examined. A micrometastasis was found in an omental lymph node and another one in a pelvic lymph node. The tumor was staged IVA according to the guidelines of the International Federation of Gynecology and Obstetrics and T_3a_N_1_M_1_ according to AJCC^3^ as previously described.^2^ The patient was B‐RAF wild type.

Treatment with Interferon alfa‐2a was offered, which the patient declined. Treatment with the oncolytic virus Rigvir^®^ was started in December 2014. The treatment with Rigvir^®^ was with administration for three consecutive days a month for 3 months; then, the medication was applied once monthly for 9 months, then every 6 weeks for a half year and then every 8 weeks for the next 6 months. In the third year of the treatment, the patient received Rigvir^®^ injection every 3 months until December 2017.

**FIGURE 4:**
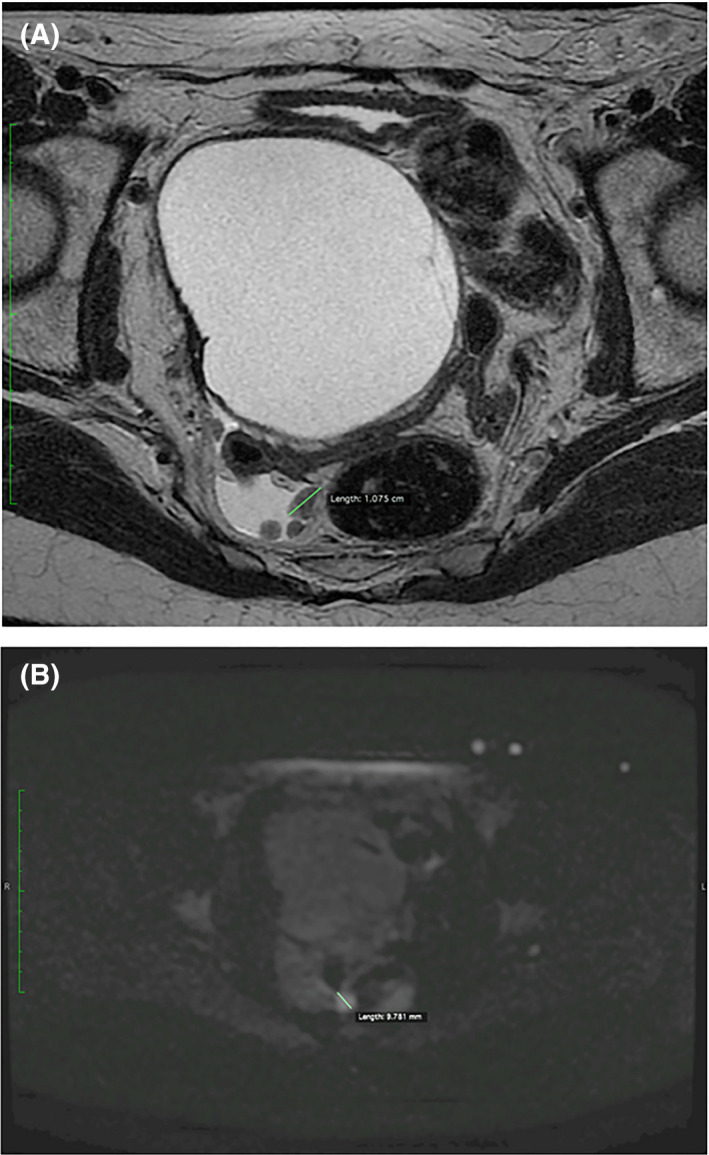
Nodes in the pelvis with restricted diffusion, largest node is 1.075 cm in diameter and ascites (magnetic resonance imaging scan performed on 22 December 2016)

**FIGURE 5:**
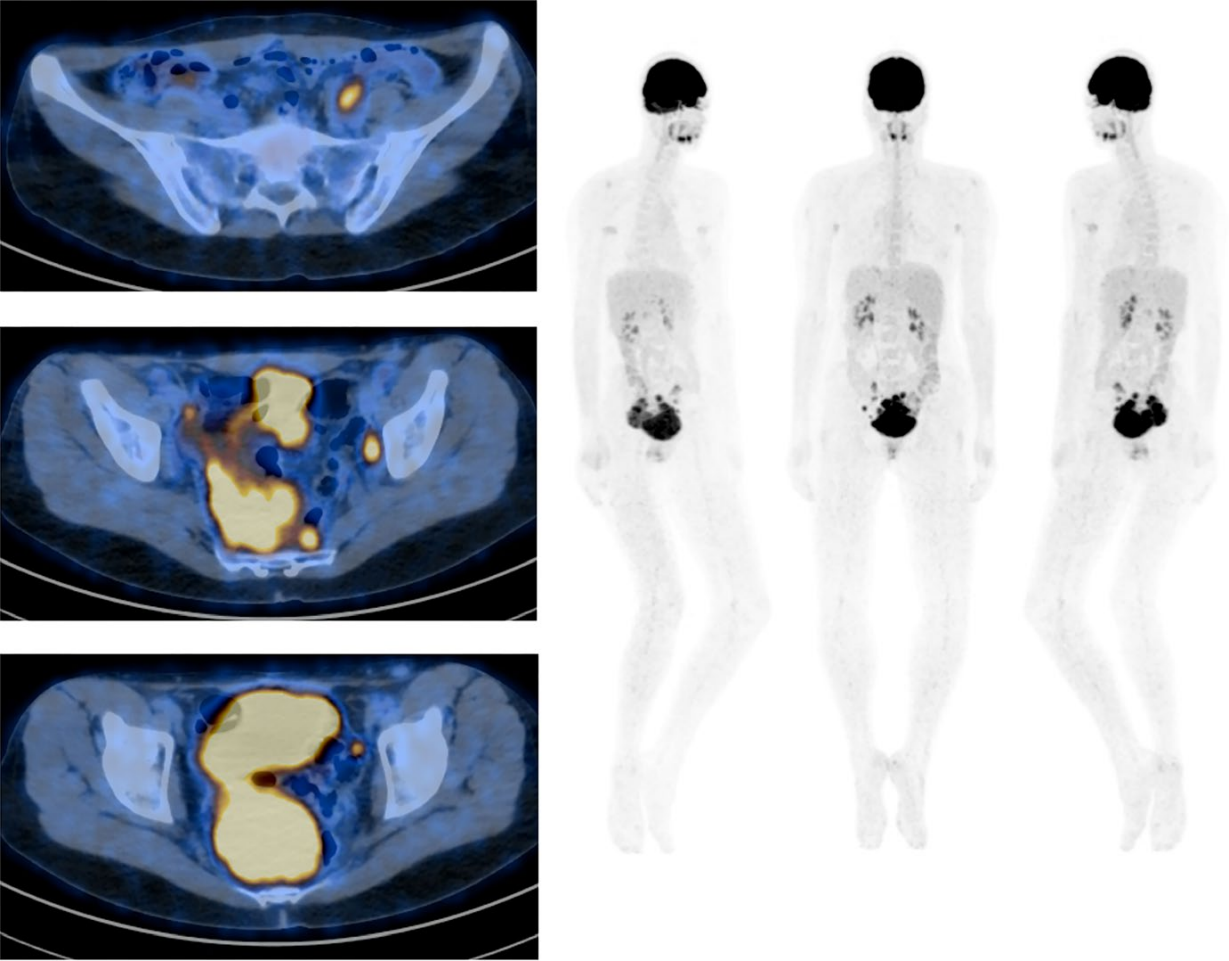
Positron emission tomography/computed tomography scan shows a big mass in the pelvis and multiple smaller nodes in the peritoneum (27 July 2019)

During the postoperative period, the formation of large transient asymptomatic bilateral lymphoceles (Figure 3I), lymph node size growth (Figure 3C‐H; Figure 4B), and ascites (Figure 4A) was found by CT or magnetic resonance imaging (MRI). In the MRI scan performed on 22 December 2016, pathological nodes with restricted diffusion were detected in the pelvis with the possibility of malignant nature (largest node 1.075 cm in axial plane) (Figure 4). On 27 July 2019, a positron emission tomography/computed tomography scan was performed. It showed a big mass in the pelvis and multiple smaller nodes that most likely are peritoneal metastases (Figure 5).

The patient underwent surgery on 14 November 2019. During surgery, melanoma dissemination was located in the abdominal cavity, mucosa, muscularis, and mesentery of the small intestine, ovary, and lymph nodes. Multiple metastatic nodes 2‐3 cm in diameter in the lesser pelvis and 2 separate metastases in urinary bladder serosa were also found. Excision of metastases, left and right hemicolectomy, and D2 lymph node dissection were performed. Pembrolizumab therapy is planned.

The patient has had no side effects or adverse events related to Rigvir^®^ treatment and maintains a good quality of life and has a current survival of 5.53 years (67 months) and progression‐free survival of 4.69 years (57 months).

## 3 DISCUSSION

This case report describes a rare tumor, a primary malignant melanoma of the uterine cervix in a 25‐year‐old female patient. The incidence of genital tract mucosal melanomas has been estimated at 2.8 cases per million females; less than 2% of them constitute cervical melanomas and almost 70% of cases occur in postmenopausal women.^1^ In addition to being a rare location of the tumor, the patient had no complaints regarding vaginal discharge, dyspareunia, and/or bleeding which would have led to an earlier diagnosis.^4^

Radical hysterectomy with pelvic lymph node dissection and partial vaginectomy have been proposed as the first‐line treatment in cases of primary malignant melanoma of the uterine cervix. Various adjuvant therapies have been used^5^, ^6^; mostly, the same chemotherapy protocols used for skin melanoma, and although mucosal melanoma is a radio‐resistant tumor, radiotherapy has also been described as an option.^7^ We are not aware of any ongoing prospective randomized trial evaluating treatment options in patients with melanomas of the female genital tract, conceivably because of the rarity of these conditions.^5^

Regardless of adjuvant or neoadjuvant therapies used so far, the overall outcome does not improve significantly. Treatment of advanced stage cutaneous melanoma has evolved and now there are target therapy regimens targeted to BRAF and KIT oncogenes,^8^, ^9^ as well as immunotherapy regimens—high‐dose Interleukin‐2, human monoclonal antibody against cytotoxic T‐lymphocyte–associated antigen 4 (CTLA‐4) that are not extensively investigated in cases of female genital tract melanoma.^10^ Lately, case reports conclude that novel immunotherapeutic agents improve survival and should be considered after testing for c‐KIT and BRAF V600E mutations in vulvovaginal melanomas.^9^

Malignant melanoma of the uterine cervix is highly aggressive tumor with high risk of local recurrence and also a risk of spreading of metastases that may appear even several years after initial diagnosis. The prognosis of primary cervical melanoma is usually poor.^1^, ^4^

The oncolytic virus Rigvir^®^ has shown effectiveness in vitro and in a retrospective study improved the clinical outcome in skin melanoma.^11^, ^12^ The present case suggests that oncolytic virotherapy might also be beneficial to study as adjuvant treatment in primary malignant melanoma of uterine cervix.

Despite any therapy used, cervical mucosal melanoma carries a high risk of recurrence, most often locally, within 3 years after initial diagnosis.^13^, ^14^ Primary malignant melanoma of cervix is usually diagnosed at FIGO stage I (41%); however, 87.5% of patients died within 3 years from diagnosis (22.9 months overall mean survival and 12 months median survival).^1^ The overall prognosis is worse since female genital tract melanoma usually spreads hematogenously at early stages.^15^ Taken together, this means the present patient shows good survival.

Another important aspect of adjuvant therapy is the quality of life. Virotherapy with Rigvir^®^ is safe, nontoxic and preserves the overall quality of life.^16^

In conclusion, considering the present patient's survival and progression‐free survival, oncolytic virotherapy with Rigvir^®^ has a good potential as adjuvant treatment in primary malignant melanoma of uterine cervix and more research is needed to define the impact on clinical outcome.

## CONFLICT OF INTEREST

AR and PA are employees of Rigvir. The rest of the authors have no conflict of interest to disclose.

## AUTHOR CONTRIBUTION

EP, ED, DK, and SD were the attending doctors of the patient. RM, SI, and TZ: carried out the histology analysis. IN and EO: carried out the radiology analysis. EP, AR, PA, and SD: made substantial contributions to acquisition of data, analysis, and interpretation of data and drafted the manuscript. All authors have read and approved the final version for publication.

## CONSENT FOR PUBLICATION

Written consent to publication has been obtained from the patient.
